# Biodegradation mechanism of 1H-1,2,4-triazole by a newly isolated strain *Shinella* sp. NJUST26

**DOI:** 10.1038/srep29675

**Published:** 2016-07-20

**Authors:** Haobo Wu, Jinyou Shen, Ruiqin Wu, Xiuyun Sun, Jiansheng Li, Weiqing Han, Lianjun Wang

**Affiliations:** 1 Jiangsu Key Laboratory of Chemical Pollution Control and Resources Reuse, School of Environmental and Biological Engineering, Nanjing University of Science and Technology, Nanjing 210094, Jiangsu Province, China

## Abstract

The highly recalcitrant 1H-1,2,4-triazole (TZ) is widely used in the synthesis of agricultural pesticide and considered to be an environmental pollutant. In this study, a novel strain NJUST26 capable of utilizing TZ as the sole carbon and nitrogen source, was isolated from TZ-contaminated soil, and identified as *Shinella* sp. The biodegradation assays suggested that optimal temperature and pH for TZ degradation by NJUST26 were 30 °C and 6–7, respectively. With the increase of initial TZ concentration from 100 to 320 mg L^−1^, the maximum volumetric degradation rate increased from 29.06 to 82.96 mg L^−1^ d^−1^, indicating high tolerance of NJUST26 towards TZ. TZ biodegradation could be accelerated through the addition of glucose, sucrose and yeast extract at relatively low dosage. The main metabolites, including 1,2-dihydro-3H-1,2,4-triazol-3-one (DHTO), semicarbazide and urea were identified. Based on these results, biodegradation pathway of TZ by NJUST26 was proposed, i.e., TZ was firstly oxidized to DHTO, and then the cleavage of DHTO ring occurred to generate N-hydrazonomethyl-formamide, which could be further degraded to biodegradable semicarbazide and urea.

1H-1,2,4-triazole (TZ), an important N-heterocyclic compound (NHC) with broad-spectrum biological activity, is widely used in the production of insecticide, herbicide, fungicide, plant growth regulator and antitumor, antivirus and antibacterial agents, *et al*.^1^,^2^,^3^. Over 0.2 million TZ derivatives have been reported in the literature, becoming extraordinarily important due to their wide-ranging biological, agrochemical and chemical properties^4^. Accompanied by their wide application, TZ and its derivatives have become the major environmental pollutants due to their highly toxic, cancerigenic and teratogenic nature^5^. In addition, TZ and its derivatives are quite recalcitrant and persistent in the soil-water system. They could not be readily hydrolyzed in the environment and showed poor volatility even at relatively high temperature^6^. Many of them have a rather long halflife in nature environment, causing serious ecological problems^7^. Thus, it is of great significance to develop a highly efficient way to remove TZ and its derivatives from the contaminated sites.

So far, physico-chemical methods such as adsorption^8^, sorption^9^, photocatalyzed mineralization^10^ and electrochemical oxidation^11^ have been developed for the degradation or transformation of TZ and its derivatives. However, physico-chemical methods have been proven to be costly and have the inherent drawbacks such as producing more recalcitrant and toxic intermediates^10^. Biological methods have turned out to be a favorable alternative, because they are highly efficient, highly selective, cost-competitive, and environmentally friendly. However, the poor biodegradability of TZ and its derivatives limits the wide application of conventional biological techniques, despite of these merits of biological techniques mentioned above. For the treatment of recalcitrant contaminants such as TZ, bioaugmentation of the conventional biological systems with specific microbes could be an effective method to achieve high removal efficiency^12^. However, species capable of degrading TZ has never been reported previously. Therefore, the isolation of species capable of degrading TZ is rather crucial for formulating an effective stratege for the bioremediation of TZ contaminants. Some species, such as *Bacillus*^12^, *Enterobacter* and *Serratia*^13^, *Kitasatospora* and *Streptomyces*^14^*, Pseudomonas*^15^, *Trichoderma*^16^ and *Shinella*^17^, have been reported to degrade the derivatives of TZ, such as triazophos, tebuconazole, ipconazole, propiconazole and nicotine. Nevertheless, some of these species could only transform the target TZ derivatives to products with TZ ring remained in the molecular structure, but without further ring cleavage^16^,^18^. Subsequent treatment was often required for complete mineralization of these intermediates. Therefore, more attention should be paid on the cleavage of TZ ring and mineralization of TZ and its derivatives, considering their potential pollution risk.

In this study, for the first time, a novel TZ-degrading strain was isolated from the soil contaminated by TZ. The effect of various factors on TZ degradation, such as pH, incubation temperature, initial TZ concentration and additional organic carbon source, was investigated. In addition, a possible degradation pathway was proposed through the identification of major intermediate metabolites. Obviously, this study will provide a new biological alternative for the bioremediation of sites contaminated by triazole, through the application of the TZ-degrading strain.

## Results

### Identification of strain NJUST26

A novel strain, which could utilize TZ as the sole carbon and nitrogen source was isolated from TZ-contaminated soil. The colony of strain NJUST26 appeared ivory white, trim on the edge, rough and raised on the surface. This strain was rod-shaped with an average size of (1.5–2.0) × (0.5–1.0) μm. This strain showed negative in Gram staining, Voges-Prokauer test, methyl red test and nitrate reduction test, but positive for oxidase and catalase.

**Figure 1:**
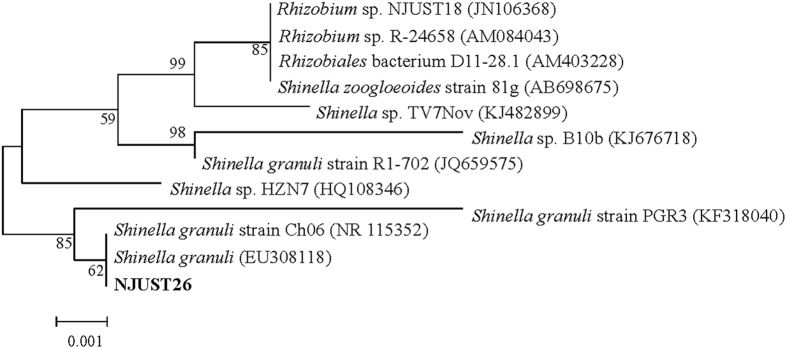
Phylogenetic tree based on 16S rDNA sequence showing the position of strain NJUST26.

In order to further identify NJUST26, the 16S rDNA sequence was determined. 16S rDNA fragment comprised of 1353 nucleotides was sequenced and submitted to the National Center for Biotechnology Information (NCBI) for BLAST analysis. The phylogenetic position of the strain NJUST26 was constructed from evolutionary distance values by the neighborjoining method, as shown in Fig. 1. The alignment indicated that NJUST26 was closely related to *Shinella granuli* strain Ch06 (NR115352) and *Shinella granuli* (EU308118), with sequence identity both as 100%. On the basis of these results, strain NJUST26 was designated as *Shinella* sp., and named after *Shinella* sp. NJUST26.

### Optimization of TZ degradation conditions

**Figure 2:**
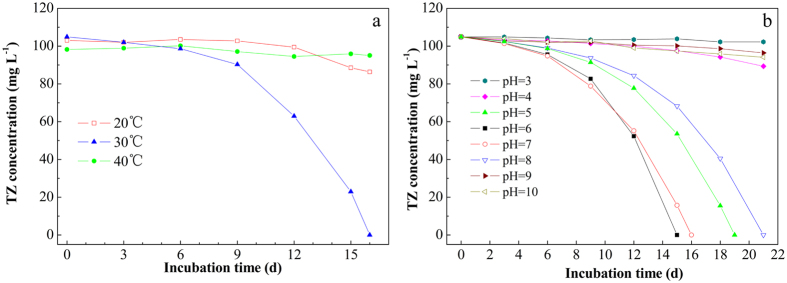
Effect of temperature (**a**) and pH (**b**) on TZ degradation by NJUST26.

Temperature is a dominant factor affecting the degradation of the recalcitrant compounds such as NHC^19^. The cell growth, metabolic and enzymatic activities can be significantly inhibited when the temperature is too high or too low. As Fig. 2a shown, TZ biodegradation by NJUST26 was found to be optimal at incubation temperature of 30 °C, with 100 mg L^−1^ TZ completely degraded within 16 d. Slow biodegradation was observed when the incubation temperature was maintained at 20 °C, while complete inhibition of TZ degradation was observed at 40 °C, which was probably due to the high sensitivity of the key enzymes involved in TZ biodegradation towards the high or low temperature. Similar phenomenon was also observed by Kang *et al*.^20^, at temperature of 45 °C, the degradation of nicosulfuron was inhibited severely. Consequently, the temperature of the follow-up experiment was controlled at 30 °C in order to achieve optimal TZ degradation performance.

For the biodegradation of xenobiotics such as TZ, the pH value of the culture media often plays an important role^21^. Figure 2b illustrated the effect of various initial pHs (3.0–10.0) of MSM on TZ degradation by NJUST26. Since phosphate buffer was added in to mineral salts medium (MSM) to offer the steady condition for the incubation of NJUST26, the pH variation was insignificant during the biodegradation process. It was observed that TZ could be effectively degraded by NJUST26 at a wide pH range of 5.0–8.0. At acid pH of 3.0 and 4.0, and alkaline pH of 9.0 and 10.0, TZ degradation could be completely inhibited. Furthermore, TZ biodegradation was optimal at an initial pH of 6.0 and 7.0, with complete TZ degradation accomplished within 15 d and 16 d, respectively. These results indicated that a slightly acidic to neutral pH might be suitable for TZ biodegradation by NJUST26.

### Effect of TZ concentration on its degradation

**Figure 3:**
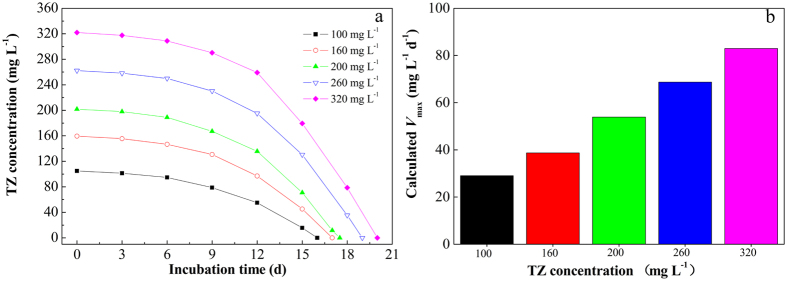
TZ biodegradation profile (**a**) and maximum volumetric degradation rates (**b**) at various initial TZ concentrations.

For inhibitory type substrate such as NHC, substrate concentration has a significant effect on the degradation efficiency^22^,^23^. As illustrated by Fig. 3a, in the MSM with TZ as the sole carbon and nitrogen source, NJUST26 could completely degrade TZ at the initial TZ concentration up to 320 mg L^−1^. At initial TZ concentrations of 100, 160, 200, 260 and 320 mg L^−1^, complete degradation was accomplished within 16, 17, 17.5, 19 and 20 d, respectively. Obvious lag phase was observed at all initial TZ concentrations, and the lag phase prolonged with the increase of initial TZ concentrations. However, as shown in Fig. 3b, with the increase of initial TZ concentrations from 100 to 320 mg L^−1^, the calculated *V*_max_ increased from 29 mg L^−1^ d^−1^ to 83 mg L^−1^ d^−1^, indicating the high tolerance and excellent degradation performance of NJUST26 towards the highly recalcitrant TZ. In the later phase of the degradation process, the degradation of TZ could be accelerated, probably due to the increased biomass. The inhibition effect of TZ and its degradation intermediates towards bacteria could be alleviated at the later phase of the degradation process.

### The effect of additional organic carbon sources on TZ degradation

**Figure 4:**
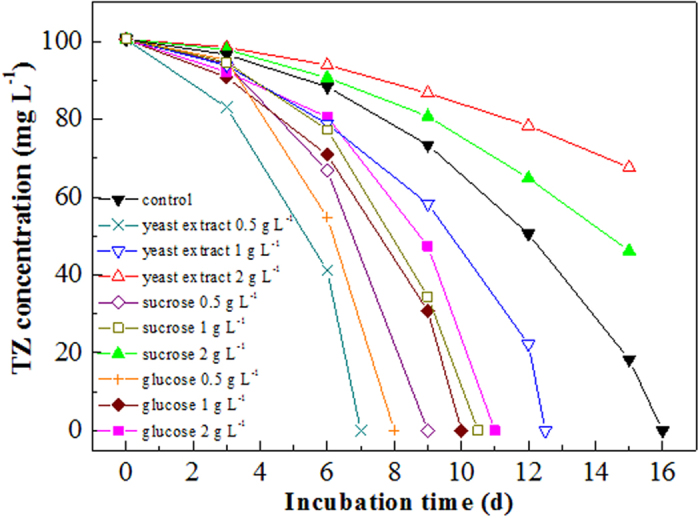
TZ biodegradation at the presence of additional organic carbon sources.

The effect of additional organic carbon sources on TZ biodegradation was investigated in this study, as shown in Fig. 4. TZ biodegradation could be dramatically accelerated after the addition of glucose, sucrose and yeast extract at low concentration. When 500 mg L^−1^ glucose, sucrose and yeast extract was added as the supplemental carbon sources, TZ biodegradation could be completed within 8, 9 and 7 d, respectively. However, with the further increase of the additional organic carbon sources, negative effect on this acceleration was observed. When 1000 mg L^−1^ glucose, sucrose and yeast extract was added, the time required for complete TZ degradation was extended to 10, 10.5 and 12.5 d, respectively. Moreover, obvious inhibition effect on TZ degradation was found when the concentration of additional sucrose and yeast extract reached 2000 mg L^−1^. However, at glucose dosage of 2000 mg L^−1^, slight acceleration in terms of TZ removal was observed.

### Identification of metabolites

**Figure 5:**
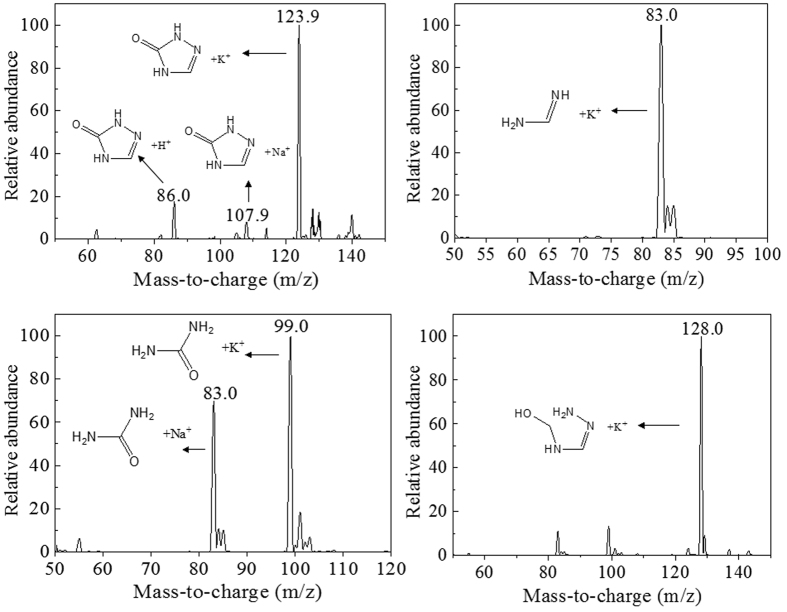
HPLC/MS analysis of metabolites during TZ degradation by NJUST26.

**Figure 6:**
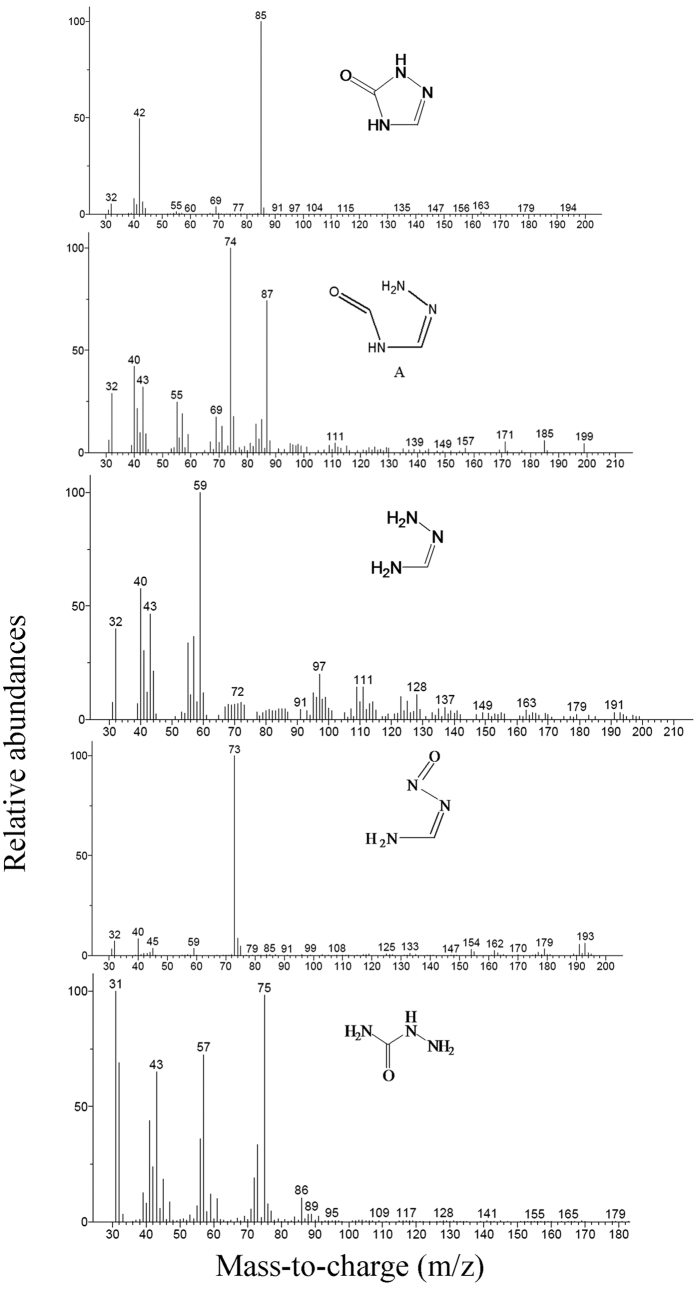
GC/MS analysis of metabolites during TZ degradation by NJUST26.

Both GC/MS and HPLC/MS were used to identify the intermediates during TZ biodegradation. Through GC/MS analysis, one of the molecular ion peaks was m/z = 85 [M+], and another main ion fragment peaks was m/z = 42 [M+-CO-NH], which was identified as the intermediate 2,4-dihydro-[1,2,4]triazol-3-one (DHTO) according to its high probability (83.1%) compare to the standard compound. Moreover, the HPLC/MS analysis showed that the most prominent protonated molecular ion peaks were found at m/z = 86 [M + H]^+^, m/z = 107.9 [M + Na]^+^, m/z = 123.9 [M + K]^+^, confirming the existence of DHTO as the main intermediate in TZ biodegradation system. Furthermore, another two intermediates were also identified through MS analysis. One intermediate, whose molecular ion peak and main ion fragment peaks were m/z = 75 [M+], m/z = 57 [M+-NH_2_-H-H] and m/z = 43 [M+-NH_2_-NH_2_] respectively, was identified as semicarbazide through GC/MS analysis based on the similarity as high as 87.4% compared to the standard compound. The other one, whose prominent protonated molecular ions were found at m/z = 83 [M + Na]^+^ , m/z = 99 [M + K]^+^, was identified as urea by HPLC/MS analysis. Five more intermediates were also detected through GC/MS or HPLC/MS analysis as shown in Table 1 and their mass spectra was shown in Figs 5 and 6.

## Discussion

*Shinella* sp. has now emerged as a promising candidate for the bioremediation of various recalcitrant pollutants. Some bacteria belonging to *Shinella* sp. have been reported to degrade nicotine^17^,^24^, 4-aminobenzenesulfonate^25^, pyridine^26^, anthracene^27^ and 3-methyl-sulfolane^28^. However, biodegradation of TZ by *Shinella* sp. has not been reported up to now. In fact, species capable of degrading TZ has never been reported previously. In this study, a novel strain, *Shinella* sp. NJUST26, which could degrade and utilize TZ as the sole carbon and nitrogen source, is reported for the first time. This result will expand the application of *Shinella* sp. in the remediation field for recalcitrant pollution.

The accelerated TZ degradation at the presence of additional carbon sources at low concentration could be attributed to the alleviation of the toxicity and inhibition effect of TZ towards NJUST26. Numerous studies have proven that the toxicity and inhibition of recalcitrant or toxic substrates could be attenuated at the presence of additional carbon sources. Degradation rates could be significantly increased by the addition of additional organic carbon sources such as yeast extract and glucose. Tobajas *et al*.^29^ found that the degradation rate of 4-chlorophenol could be dramatically increased by the addition of glucose. Chen *et al*.^30^ studied the biodegradation of methyl *tert*-butyl ether and found that the individual addition of yeast extract, beef extract and tryptone exhibited stimulatory effect on the degradation of methyl *tert*-butyl ether. It was demonstrated that glucose could induce the formation of monooxygenase required for the transformation of many recalcitrant compounds such as quinoline. In addition, the consumed NADH necessarily used for the biological process could be efficiently regenerated through glucose oxidation^31^, Although the mechanisms for the cometabolic degradation of various pollutants by microbes were rather complicated, the first step was considered to be the utilization of easily degradable substrate by microbes and the production of catabolic enzymes with broad substrate specificity, which could be used for the degradation of recalcitrant substrate^32^. What’s more important, the addition of organic carbon source often results in the increase of biomass, which is beneficial for the degradation of various recalcitrant substrates. However, the acceleration of TZ degradation by the additional carbon sources was concentration dependent. Overhigh dosage of the additional carbon sources often resulted in the delay of TZ biodegradation, which could be attributed to the competition of nutrition such as oxygen and trace elements, especially at the presence of abundant additional carbon sources^23^.

**Figure 7:**
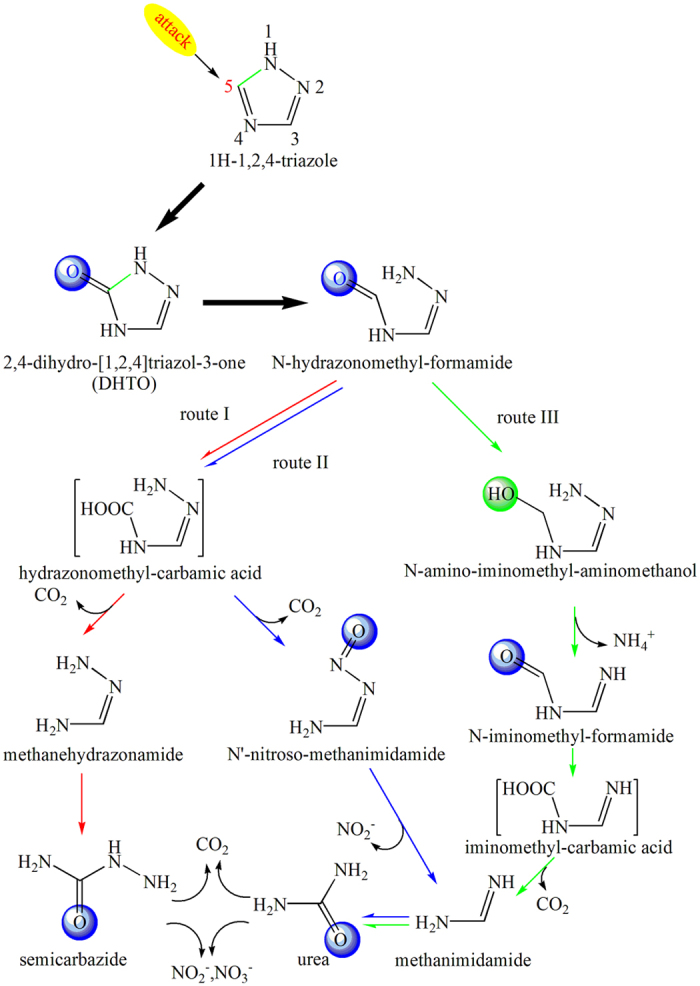
Proposed metabolic pathway of TZ by NJUST26.

Based on the identified DHTO, semicarbazide, urea, as well as other intermediates presumed to be involved in TZ degradation, the metabolic pathways of TZ was proposed, as demonstrated in Fig. 7. TZ was firstly oxidized to DHTO through mono-oxidation, then the cleavage of DHTO ring occurred and DHTO was further transformed to N-hydrazonomethyl-formamide. Afterwards, N-hydrazonomethyl-formamide was further transformed through three routes. In route I, it was oxidized to hydrazonomethyl-carbamic acid, which was futher decarboxylized to form methanehydrazonamide through the cleavage of the C-N bond. Then methanehydrazonamide was further oxidized to semicarbazide, which could be subsequently mineralized. In route II, hydrazonomethyl-carbamic acid was first generated as described above, then oxidation reaction happened to both ends of its molecule, with the formation of N′-nitroso-methanimidamide. Then the nitroso group on N′-nitroso-methanimidamide was eliminated, resulting in the formation of methanimidamide, with NO_2_^−^ released simultaneously. In route III, hydration reaction occurred on N-hydrazonomethyl-formamide, with the formation of N-amino-iminomethyl-aminomethanol, which could be further oxidized and deaminated to iminomethyl-carbamic acid, with NH_4_^+^ released simultaneously. Then iminomethyl-carbamic acid could be transformed to methanimidamide through decarboxylation reaction. Methanimidamide generated from route II and route III was further mono-oxidized to urea, which could be mineralized further. In an electrochemical system for TZ oxidation, attack of HO**·** radicals occurred on the TZ ring, leading to the cleavage TZ ring and formation of N-methylene-methanehydrazonamide, followed by the further oxidization by HO**·** radical to give N-amino-iminomethyl-aminomethanol, which could be further oxidized to organic acids and inorganic anions or CO_2_^11^. During the electrochemical oxidation process, the cleavage product of TZ was found to be N-methylene-methanehydrazonamide, which was different from this study. In a photocatalysis system, the ring cleavage step was similar to that in electrochemical oxidation system, with N-methylene-methanehydrazonamide as the main intermediate, which would be further degraded to refractory cyanuric acid^10^. In this study, a new degradation pathway was proposed, expanding the knowledge in terms of the degradation of refractory TZ.

According to the frontier-electron theory, the attack reaction in TZ structure preferentially occurred in the positions where the atom bore the higher frontier electron density on the basis of quantum chemistry. According to calculated frontier electron densities for all atoms in the TZ structure, the atom with the greater frontier electron density was the N1 (0.515) and C5 (0.470) atoms, where became the active sites at the presence of some oxidant^10^. Thus, it could be assumed that the attack of oxidant firstly occurred on N1 and C5 atoms, which was confirmed from the identification of N-hydrazonomethyl-formamide through the GC/MS analysis. Similar results were observed in a biodegradation system for tebuconazolc, triazole ring in tebuconazol was found to be cleaved at N1 position^16^. What’s more, It has been proposed that in both electrochemical oxidation and photocatalysis systems, the attack of HO**·** radicals on the TZ ring led to ring cleavage in N1 and C5 position^10^,^11^. Furthermore, during the cleavage of TZ ring in other TZ derivatives, such as tebuconazol and triazophos, the break of the bond connecting N1 and C5 in TZ ring was often considered as the first step of the ring cleavage^16^,^18^,^33^, which was consistent with this study.

## Conclusion

In this paper, a strain capable of utilizing 1H-1,2,4-triazole (TZ) as sole carbon and nitrogen source was firstly isolated and identified as *Shinella* sp. NJUST26. TZ could be optimally degraded at temperature of 30 °C and pH of 6.0–7.0. With the increase of initial TZ concentration, maximum volumetric degradation rate increased, indicating high tolerance of NJUST26 towards TZ. TZ biodegradation could be accelerated through the addition of glucose, sucrose and yeast extract at relatively low dosage. Eight main intermediates, including 2,4-dihydro-[1,2,4]triazol-3-one, semicarbazide and urea, were identified through GC/MS and HPLC/MS analysis, with the biodegradation pathway of TZ by NJUST26 proposed.

## Materials and Methods

### Growth media and cultivation condition

Analytical-grade TZ (99.0% purity) was purchased from Aladdin Industrial Corporation (Shanghai, China). Enrichment and isolation of the species capable of degrading TZ was performed in MSM containing 3.06 g L^−1^ Na_2_HPO_4_·12H_2_O, 0.76 g L^−1^ KH_2_PO_4_, 0.2 g L^−1^ MgSO_4_·7H_2_O, 0.25 g L^−1^ CaCl_2_, 10 mL L^−1^ trace elements solution SL-4^34^ and TZ at desired concentrations. Store culture was maintained by periodical transfer onto LB plates supplemented with 100 mg L^−1^ TZ and was stored at 30 °C for further study. For long-term maintenance, TZ degrading strain was stored in 20% glycerol at −80 °C in an ultra-low temperature freezer.

The cultures were incubated in liquid MSM on a rotary shaker at 180 rpm and 30 °C. The biodegradation experiment was conducted using a series of 100 mL Erlenmeyer flasks as batch reactors. Before inoculation, 50 mL liquid MSM supplemented with TZ at desired concentrations was transferred to each Erlenmeyer flask and autoclaved at 121 °C for 30 min.

### Enrichment and isolation of TZ-degrading strain

The soil samples were collected from the sites contaminated by TZ in Jintan Fengdeng Environmental Technology Service Co. Ltd, which was located in Jiangsu Province, China. Twenty soil samples were randomly collected in sterile containers from the sites 5–10 cm below the land surface. The collected samples were air-dried, and then mixed and ground in an agate mortar to pass through a 2-mm nylon sieve. About 10 g ground soil sample described above was inoculated into 250 mL Erlenmeyer flasks containing 100 mL liquid MSM containing 100 mg L^−1^ TZ, and incubated on a rotary shaker at 180 rpm and 30 °C. 3 hours later, 1 mL supernatant liquor was transferred into 50 mL fresh MSM containing 30 mg l^−1^ TZ. After 15-day incubation, 1 mL culture was transferred to the same fresh MSM for another round of incubation for the enrichment of TZ-degrading bacteria. Then the culture were further enriched and acclimated through successive transfer to fresh MSM, with TZ concentration further increased to 50 and 100 mg L^−1^, respectively. Subsequently, the culture was serially diluted and streaked onto LB plates supplemented with 100 mg L^−1^ TZ. About 1 week later, single colonies were picked and re-streaked for purification three times. Then, pure colonies were transferred to MSM containing 100 mg L^−1^ TZ to investigate their ability of degrading TZ. Finally, one purified strain capable of degrading TZ was obtained and named after NJUST26.

### Strain identification

The morphological, physiological and biochemical tests were used to characterize the strain NJUST26. The strain was further identified by 16S rDNA sequence analysis. Genomic DNA of NJUST26 was extracted from fresh cells grown in the liquid LB, using the TaKaRa MiniBEST Bacteria Genomic DNA Extraction kit Ver.3.0 (TaKaRa Bio, Japan) according to the manufacturer’s instruction. The extracted 16S rDNA was amplified by polymerase chain reaction (PCR) using primers 27F (50-AGAGTTTGAT CCTGGCTCAG-30) and 1492R (50-TACCTTGTTACGACTT-30). Reaction mixture contained each primer (0.4 μL), deoxy-nucleotide triphosphates (dNTPs, 2 μL), 10× reaction buffer (3 μL), Taq polymerase (0.15 μL), DNA template (0.2 μL), Mg^2+^ solution (1.8 μL), and sterile water (22.05 μL) to achieve a final volume of 30 μL. PCR was performed in a mastercycler gradient PCR thermocycler (Bio-Rad S1000TM, USA) with an initial denaturation at 94 °C for 10 min, followed by 30 cycles at 94 °C for 30 sec, 55 °C for 30 sec, and 72 °C for 1.5 min, followed by a final extension performed at 72 °C for 10 min. The target DNA fragment was purified and then sent for sequencing according to the manufacturer’s instruction. The sequence was deposited in the GenBank database under accession No. KP890249. Nucleotide sequence similarity was analyzed using BLAST (National Center for Biotechnology Information Databases).

### TZ biodegradation assay

The pure strain NJUST26 was inoculated in LB medium supplemented with 100 mg L^−1^ TZ and incubated at 30 °C and 180 rpm until the bacteria grew into the logarithmic phase (about 48 h after inoculation). The bacteria were harvested by centrifugation at 6,000 × g for 5 min. The deposition was resuspended, washed twice and diluted with fresh MSM to an OD_600_ of about 2.0 finally. The bacterial suspension was immediately employed as the inocula in the following biodegradation experiment, with inoculum size controlled at 2%.

To investigate the effect of incubation temperature on TZ degradation, 50 mL MSM containing 100 mg L^−1^ TZ were inoculated with pure NJUST26 inocula and incubated at temperature of 20 °C, 30 °C and 40 °C, respectively, at initial pH of 7.0. To study the effect of initial pH on TZ degradation, the same experiment was carried out except that the incubation temperature was stabilized at 30 °C and the initial pH of the medium was adjusted to 3.0–10.0 under buffer system through changing dosage of Na_2_HPO_4_·12H_2_O or KH_2_PO_4_ or adding HCl or NaOH (1 mol L^−1^), respectively. To study the effect of initial TZ concentration on its degradation, pure NJUST26 inocula was inoculated into the MSM containing 100–320 mg L^−1^ TZ, at initial pH of 7.0 and incubation temperature of 30 °C. MSM containing 100 mg L^−1^ TZ, supplemented with different concentrations of glucose, sucrose and yeast extract (500 mg L^−1^, 1000 mg L^−1^ and 2000 mg L^−1^, respectively) was used to investigate the effect of the additional carbon sources on TZ degradation.

### Analytical and calculation methods

Before analysis, water samples were filtered immediately after sampling through a 0.22 μm membrane. TZ concentration was quantified by HPLC (LC-20AT, SHIMADZU, Japan) with a Inertsil^®^ ODS-SP column (5 μm, 4.6 × 250 mm) and a diode array detector through authentic standard. The mobile phase was a mixture of 10% methanol and 90% ultrapure water pumped at a flow rate of 1.00 mL min^−1^. The sample injection volume was 10 μL. The analysis was performed at 195 nm, with column temperature of 40 °C. The intermediates of TZ biodegradation were analyzed by GC/MS (Agilent 7890A GC/5975C MSD, HP-5MS column, USA) and HPLC/MS (Agilent 6410 HPLC/MS, USA). For GC/MS analysis, ethyl acetate was chosen as extraction agent for TZ and its biodegradation products in the water sample because of its relatively high polarity, and the obtained organic solution was concentrated by a rotary evaporator at 80 °C and redissolved in methanol. The column temperature program for GC/MS was isothermal at 40 °C for 2 min and then increased from 40 °C to 280 °C with an increment of 10 °C min^−1^ and kept isothermal for 3 min at last. The ion source temperature and quadrupole temperature were 250 °C and 150 °C, respectively, and the electron energy was 70 eV. The condition for HPLC-MS was as follows: column type were Agilent Eclipse Plus C18 column (3.5 μm, 2.1 × 150 mm), methanol and ultrapure water (10:90, V:V) was used as mobile phase at the flow rate of 0.2 mL min^−1^. MS analyses were performed in +ESI mode with an Agilent G6410B Triple Quad Mass Spectrometer. The ESI and the ion-trap analyzer MS parameters were optimized to reach the best sensitivity for intermediates.

Triazole biodegradation kinetics was described and modelized with Gompertz equation, according to Shen *et al*.^35^:


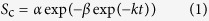


where *S*_c_ is the triazole consumed (mg L^−1^), *t* is the incubation time (d), and *α* (mg L^−1^)*, β* (mg L^−1^)*, k* (d^−1^) is the fitting parameters of the Gompertz model. The three fitting parameters were calculated through the modeling of *S*_c_ data with *t*. Maximum volumetric degradation rate (*V*_max_, mg L^−1^ d^−1^) was then calculated through the equation:


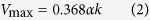


## Additional Information

**How to cite this article**: Wu, H. *et al*. Biodegradation mechanism of 1H-1,2,4-triazole by a newly isolated strain *Shinella* sp. NJUST26. *Sci. Rep.* **6**, 29675; doi: 10.1038/srep29675 (2016).

## Figures and Tables

**Table 1:**
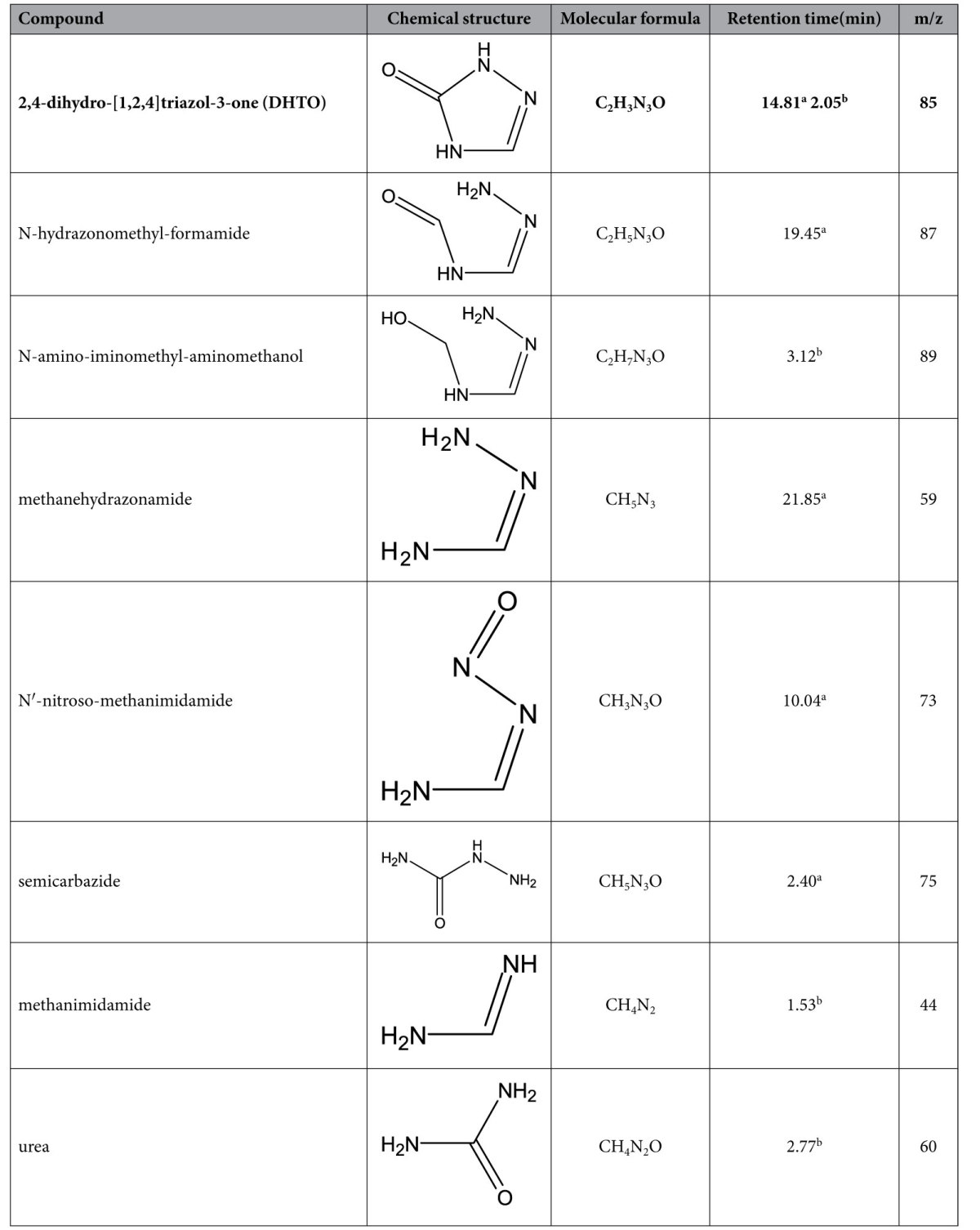
Intermediates during TZ degradation identified by GC/MS and LC/MS.

^a^Obtained by GC/MS.

^b^Obtained by HPLC/MS.
